# Supplementary figures and images for: SEQing: web-based visualization of iCLIP and RNA-seq data in an interactive python framework
Source: BMC Bioinformatics. 2020 Mar 18;21:113. doi: 10.1186/s12859-020-3434-9 (PMC7079501; doi:10.1186/s12859-020-3434-9)

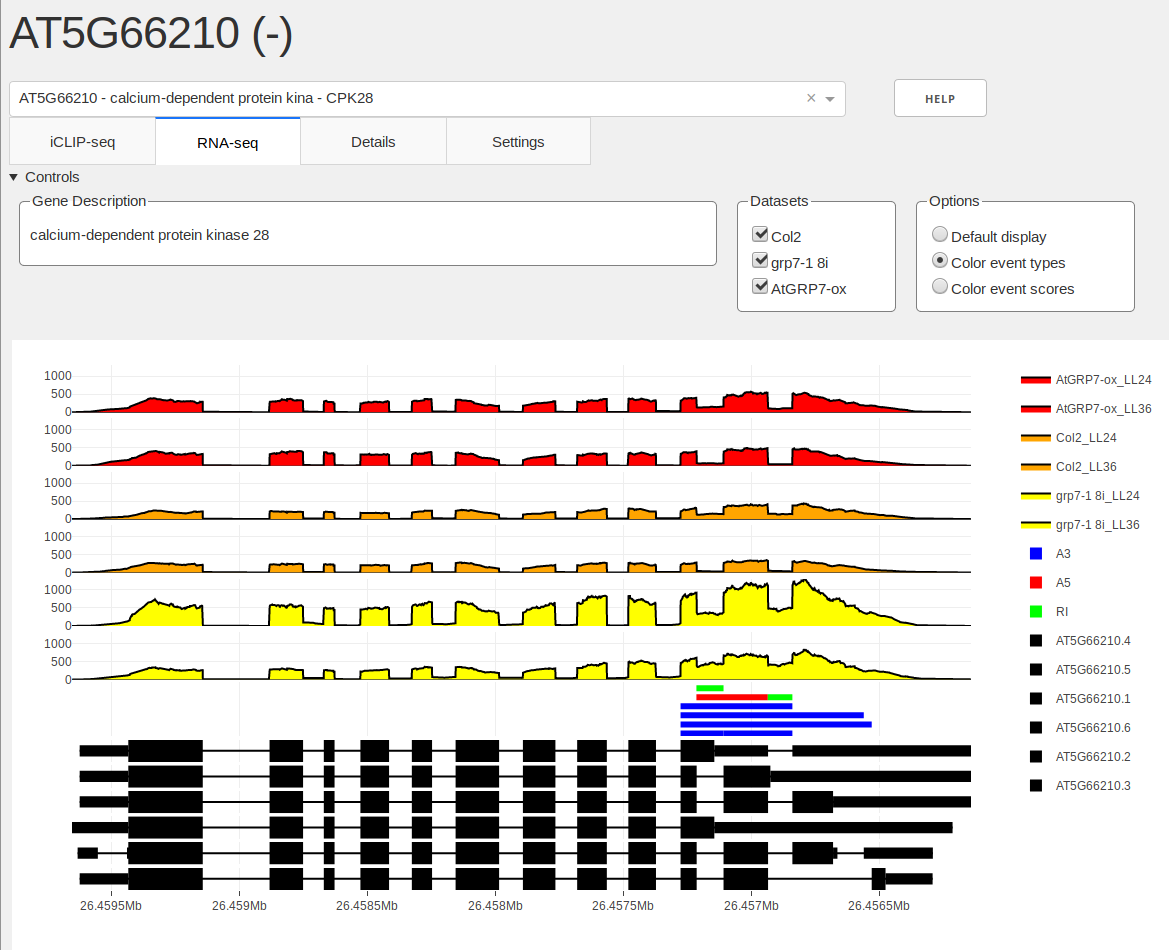

Supplement: Supplementary file 1 — Additional file 1 Source code and sample data for SEQing. The Python code and samples of Arabidopsis thaliana iCLIP (GSE99427) and RNA-seq (GSE99615) data used to start the sample dataset dashboard. [file 12859_2020_3434_MOESM1_ESM.zip › SEQing-master/SEQing_RNA_sample.png]

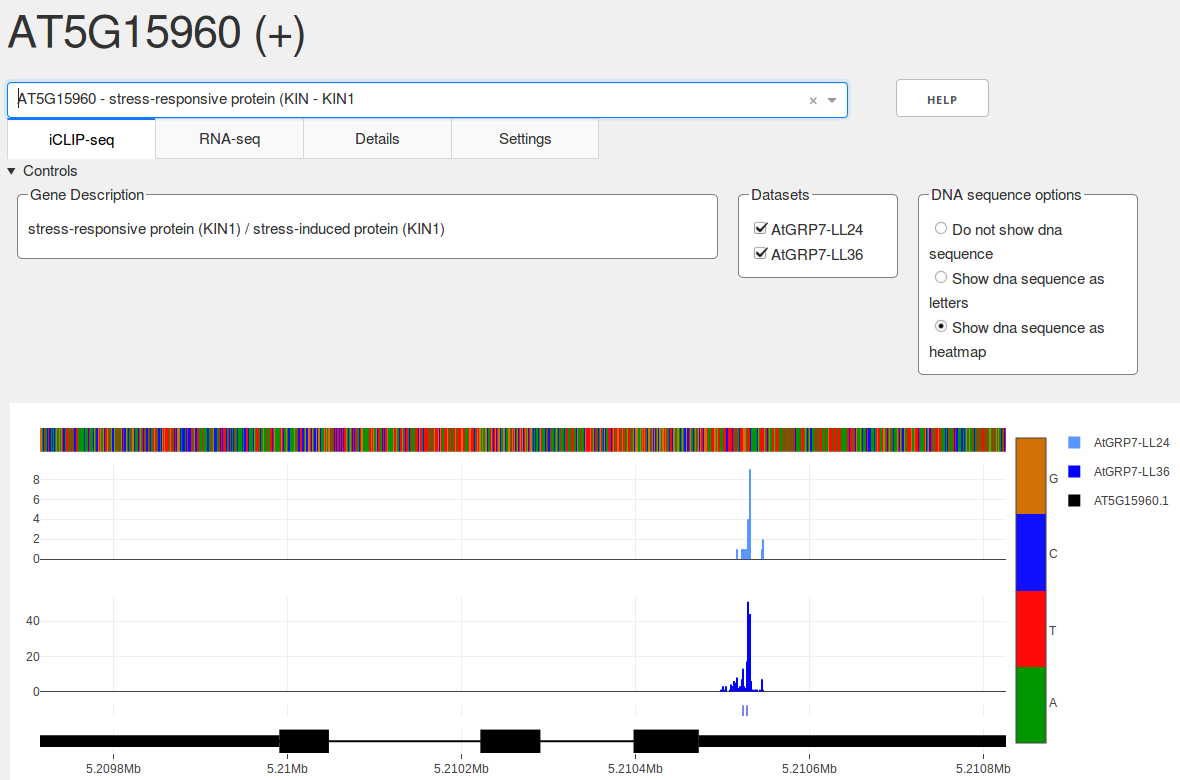

Supplement: Supplementary file 1 — Additional file 1 Source code and sample data for SEQing. The Python code and samples of Arabidopsis thaliana iCLIP (GSE99427) and RNA-seq (GSE99615) data used to start the sample dataset dashboard. [file 12859_2020_3434_MOESM1_ESM.zip › SEQing-master/SEQing_iCLIP_sample.PNG]

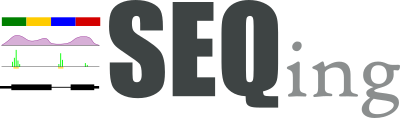

Supplement: Supplementary file 1 — Additional file 1 Source code and sample data for SEQing. The Python code and samples of Arabidopsis thaliana iCLIP (GSE99427) and RNA-seq (GSE99615) data used to start the sample dataset dashboard. [file 12859_2020_3434_MOESM1_ESM.zip › SEQing-master/Seqing.png]
